# Omics Methods in Toxins Research—A Toolkit to Drive the Future of Scientific Inquiry

**DOI:** 10.3390/toxins14110761

**Published:** 2022-11-04

**Authors:** Joseph D. Romano

**Affiliations:** 1 Center of Excellence in Environmental Toxicology, University of Pennsylvania, Philadelphia, PA 19104, USA; 2 Institute for Biomedical Informatics, University of Pennsylvania, Philadelphia, PA 19104, USA; 3 Department of Biostatistics, Epidemiology & Informatics, University of Pennsylvania, Philadelphia, PA 19104, USA

A major trend in biomedical research has been an ideological shift away from studying individual components of an organism or biological process in isolation, and towards how those components function collectively, forming the basis of the field now known as systems biology [1]. Virtually all domains in biology and medicine have been influenced by the systems approach to scientific discovery, and toxinology is no exception. Toxins exist in complex living systems, and their activities influence other living systems, mediated by many biomolecules, metabolites, and cofactors. Omics methods are one of the main classes of tools for studying toxins in a systems biology context. Omics methods can be used to analyze the entire collection of a group of components simultaneously [2]. Genomics techniques look at the entire collection of genes in a living system, transcriptomics looks at the entire collection of mRNA transcripts, metabolomics looks at all the metabolites, and so on. Omics also includes the secondary analysis of the data generated through these experiments, as well as the storage/cataloging of these data [3].

In recent decades, we have seen diverse examples of omics methods used to great effect in toxinology. This includes venomics [4]—further divided into venom proteomics, venom metabolomics, and others—where researchers identify and analyze the complete molecular makeup of a species’ venom or the genes that encode venom proteins. However, although venomics has been particularly prolific, virtually every class of toxin has been subjected to omics techniques via high-throughput sequencing, fractionation/purification, bioinformatics analysis, and other protocols that collectively define what “omics methods” are. As an exercise, you can take the name of any class of toxins and search Google Scholar or PubMed for “[toxin type] omics” and you can expect to find at least a few comprehensive reviews along with many pages of targeted investigations, almost all of which have been conducted in the past ten years.

Until now, there have been a few major “styles” of omics studies in toxins research, irrespective of the types of toxins under consideration. The first is to catalog toxins consisting of mixtures. As mentioned already, venomics provides a prime example of this, but other classes of toxins—such as phytotoxins [5] and mycotoxins [6]—do the same. Constructing taxonomies of toxin mixtures can be a daunting task, especially as some toxins consist of hundreds of distinct compounds, any one of which can present unique challenges for isolation. In the case of genomics, this often takes the form of functional genomics research, where the organism producing the toxin(s) has its genome sequenced, and genes encoding toxins (or structures that synthesize toxins) are identified [7], but it can also be used to explain how toxins and toxin synthesis systems evolved. The next logical step beyond cataloging toxins with omics is using omics methods to understand the context and mechanisms of how they are synthesized.

Once a toxin’s composition and synthesis are known, researchers can then use other omics techniques to learn their function once they are delivered to their biological target. In the case of mixtures, the overall function is often greater than the sum of its parts: different components work together to incite activities not seen in any of them on their own, underscoring the importance of a systems biology approach. The responsible processes can be heavily influenced by the target organisms’ metabolome [8], proteome [5], and microbiome [9]. The coming years will almost certainly expose other -omes as key players for toxin response, such as the target organism’s lipidome and immunoproteome.

Clearly, omics methods have become established in the routine practice of toxins research and have opened new horizons for how we understand toxin form and function. Given the vast diversity of natural toxins, there is plenty to still be done. However, while researchers continue to apply existing omics methods to new, understudied species and contexts, we call on scientists to continually develop new types of omics applications in toxinology that will keep the field at the forefront of biotechnology. Furthermore, innovation will necessitate using existing omics methods to answer new types of questions. For example, how can omics research on humans teach us about the effects of toxins on our own health? Additionally, how can we leverage multi-omics (linking several omics techniques in parallel or tandem) [10] to improve upon existing work? Toxinology has always kept up with cutting-edge trends in biotechnology, and if it continues to do so, questions like these will soon become standard in applied and clinical toxinology. Omics methods will not replace or invalidate basic research in the study of toxins, but they certainly will be a mandatory tool in every toxinologist’s toolbox.

## Data Availability

Not applicable.
